# Supplementary material for: Symmetric exchange of multi-protein building blocks between stationary focal adhesions and the cytosol
Source: eLife. 2014 Jun 3;3:e02257. doi: 10.7554/eLife.02257 (PMC4040925; doi:10.7554/eLife.02257)
Supplement: Supplementary file 2. — List of primers. All primers are shown in a 5′ to 3′ orientation. DOI: http://dx.doi.org/10.7554/eLife.02257.011 [file elife02257s002.docx]

| eGFP-meGFP-FP | TGAGCACCCAGTCCAAGCTGAGCAAAGACCCCA |
| --- | --- |
| eGFP-meGFP-RP | TGGGGTCTTTGCTCAGCTTGGACTGGGTGCTCA |
| mKate2 FP1 | Ccaccggtcgccaccatggtgagcgagctgattaagg |
| mKate2 RP1 | ggCTCGAGATCTGAGTCCGGAtctgtgccccagtttgctagg |
| mKate2 C1 FP1 | GCGCaccggtGCAGGTGCTGGAATGGTGAGCGAGCTGATTAAGG |
| mKate2 C1 RP1 | GCGCtccggaTCTGTGCCCCAGTTTGCTAGG |
| mKate2 N1 FP1 | GCGCaccggtcgccaccATGGTGAGCGAGCTGATTAAGG |
| mKate2 N1 RP1 | GCGCtccggaCCGGCTCTGTGCCCCAGTTTGCTAGG |
| Don1-FP | tatAGATCTagcgcgggcGGAAAGAAAAATAGAAAGGGAAAAGAAAAT |
| Don1-RP | tatGAATTCgcccgcgctCGTAAAACTTAATTCTTGTCTAGATGGATGAA |
| meGFP-FP | ataGTCGACcaATGGTGAGCAAGGGCGAGGAGC |
| meGFP-RP | ATAggatccTTACTTGTACAGCTCGTCCATGCCGA |
| Parvin C1 FP1 | ggCTCGAGctatggccacctccccgcagaagtcg |
| Parvin C1 RP1 | ccGAATTCtcactccacgttacggtacttgg |
| CAS C1 FP1 | cgcgcgCTCGAGcgatgaaccacctgaacgtgctggc |
| CAS C1 RP1 | gcgcgcGAATTCtcaggcggctgccagctg |
| Csk N1 FP1 | ccGTCGACatgtcagcaatacaggccgcctgg |
| Csk N1 RP1 | ccGGATCCgccaggtgcagctcgtgggttttg |
| FAK C1 FP1 | ggagatctgggATGGCAGCTGCTTACCTTGACC |
| FAK C1 RP1 | ccgtcgacTCAGTGTGGTCTCGTCTGCCC |
| ILK C1 FP1 | ggCTCGAGctatggacgacattttcactcagtgc |
| ILK C1 RP1 | ccGAATTCctacttgtcctgcatcttctcaagg |
| PINCH-FP2 | tatAGATCTgcaggttccgctggtATGGCCAACGCCCTGGCCAGCGCC |
| PINCH-RP2 | TATgtcgacTTATTTCCTTCCTAAGGTCTCAGCTAGTTTCTTAAGTCTTTTCTTCAGCTCC |
| Talin C1 FP2 | gcgcGAATTCgatggttgcactttcactgaagatcagc |
| Talin C1 RP2 | gcgcGTCGACttagtgctcatctcgaagctctgaagg |
| mCitrine-FseI FP | ccggactcagatctcgagctcaagcttcgaattctgcagGGCCGGCCctgcagtcgacggtaccgcgggcccg |
| mCitrine-FseI RP | cgggcccgcggtaccgtcgactgcagGGCCGGCCctgcagaattcgaagcttgagctcgagatctgagtccgg |
| Tensin-FP | ATAgtcgacATGAGTGTGAGCCGGACCATGGAG |
| Tensin-RP | ataGGTACCttaTCTCTTTTGGCCGGCATTCAGCATGAC |
| VASP N1 FP1 | ccgaattcATGAGCAGCGAGACGGTCATCTG |
| VASP N1 RP1 | ccggatccgcGGGAGAACCCCGCTTCCTCAGC |
| Vinculin FP1 | ggtccggaagtgctggtagtgctggtATGCCAGTGTTTCATACGCGCAC |
| Vinculin RP1 | ccgtcgacCTACTGGTACCAGGGAGTCTTTCTAACCCA |
| meGFP-dSH2 FP1 | TACAAGTCCGGACTCAGATCCGAGCTCAAGCTTAAGAGCA |
| meGFP-dSH2 RP1 | TGCTCTTAAGCTTGAGCTCGGATCTGAGTCCGGACTTGTA |
